# Collectin CL-LK Is a Novel Soluble Pattern Recognition Receptor for *Mycobacterium tuberculosis*

**DOI:** 10.1371/journal.pone.0132692

**Published:** 2015-07-14

**Authors:** Anthony Troegeler, Geanncarlo Lugo-Villarino, Søren Hansen, Voahangy Rasolofo, Maiken Lumby Henriksen, Kenichiro Mori, Katsuki Ohtani, Carine Duval, Ingrid Mercier, Alan Bénard, Jérome Nigou, Denis Hudrisier, Nobutaka Wakamiya, Olivier Neyrolles

**Affiliations:** 1 Centre National de la Recherche Scientifique, Institut de Pharmacologie et de Biologie Structurale, Toulouse, France; 2 Université de Toulouse, Université Paul Sabatier, Institut de Pharmacologie et de Biologie Structurale, Toulouse, France; 3 Department of Cancer and Inflammation Research, Institute of Molecular Medicine, University of Southern Denmark, Odense, Denmark; 4 Mycobacteria Unit, Pasteur Institute in Antananarivo, Antananarivo, Madagascar; 5 Department of Microbiology & Immunochemistry, Asahikawa Medical University, Asahikawa, Japan; The Ohio State University, UNITED STATES

## Abstract

Understanding the molecular components of immune recognition of the tuberculosis (TB) bacillus, *Mycobacterium tuberculosis*, can help designing novel strategies to combat TB. Here, we identify collectin CL-LK as a novel soluble C-type lectin able to bind *M*. *tuberculosis*, and characterize mycobacterial mannose-capped lipoarabinomannan as a primary ligand for CL-LK. Mice deficient in CL-K1, one of the CL-LK subunits, do not display altered susceptibility to *M*. *tuberculosis*. However, we found that the amount of CL-LK in the serum of patients with active TB is reduced, compared to that in controls, and correlates inversely to the magnitude of the immune response to the pathogen. These findings indicate that CL-LK might be of interest for future diagnostic and treatment monitoring purposes.

## Introduction

*Mycobacterium tuberculosis*, the agent of tuberculosis (TB) in humans, is still responsible for nearly 1.5 million deaths and over 8 million new cases every year in the world [1]. Control and possible eradication of TB in the decades to come will require shorter and more effective treatments, a vaccine better than BCG, together with improved tools for diagnostic and treatment monitoring. A better understanding of the immune pathways involved in host defense against *M*. *tuberculosis* might help design better vaccination strategies, and identify novel biomarkers.

Early recognition of *M*. *tuberculosis* and mounting of an appropriate response by the immune system relies on a range of membrane-bound and soluble receptors, the so-called pattern recognition receptors, including Toll-like receptors (*e*.*g*. TLR-2) and C-type lectins (*e*.*g*. DC-SIGN) [2]. Soluble immune receptors for *M*. *tuberculosis* include surfactant proteins A (SP-A) and D (SP-D), and other collectins, such as the mannose-binding lectin (MBL) [3,4]. Collectins are homomeric or heteromeric proteins containing a collagen-like region and a C-type lectin domain separated by an α-helical coiled-coil region [5]. In *M*. *tuberculosis*, SP-A and SP-D bind various glycoconjugates, including cell envelope mannose-capped lipoarabinomannan (ManLAM) [6,7]. Several *in vitro* studies showed that SP-A and-D modulate immune response to the TB bacillus, including binding and phagocytosis by host macrophages and epithelial cells, intracellular trafficking and phagosome-lysosome fusion within infected host cells, cytokine production and T cell activation [3,4]. However, SP-A-, SP-D and SP-A/D-deficient mice are not more susceptible to *M*. *tuberculosis* than their wild-type counterparts [8].

Collectin-11 (CL-11, alias CL-K1) is a soluble protein expressed primarily in the adrenal gland, kidney, and liver, as well as in other organs, such as the lung, although to a lesser extent [9,10]. It binds to apoptotic cells and distinct pathogen species (*e*.*g*. *Escherichia coli*, *Candida albicans*, influenza virus, but not *Listeria monocytogenes* or *Pseudomonas aeruginosa*), leading to complement activation via mannan-binding lectin-associated serine proteases (MASPs) [11,12]. Recombinant CL-K1 mostly forms monomers and dimers of subunits composed of three polypeptide chains; however, *in vivo*, native CL-K1 forms more complex heteromeric structures, ranging from dimers to hexamers of subunits in tight association with another structurally-related collectin, collectin L1 (CL-L1) [13], forming what is now designated CL-LK [14].

Since CL-K1 recognizes mannose [11], and given that the mycobacterial cell envelope is rich in mannose-containing glycoconjugates, including glycoproteins, glycolipids and lipoglycans, we asked whether CL-K1, either in recombinant or native CL-LK form, could recognize *M*. *tuberculosis* and influence immunity to *M*. *tuberculosis*. Using a combination of *in vitro* and *in vivo* approaches, here we report that, while CL-K1 binds to the bacillus through its ManLAM component, mice deficient for this collectin do not display any increased susceptibility or altered immunopathology related to *M*. *tuberculosis* infection. Notwithstanding, an assessment of the amount of circulating CL-LK in patients with TB, as compared to in healthy infected and non-infected control individuals, revealed an inverse correlation to the magnitude of the immune response to *M*. *tuberculosis*, suggesting that CL-LK might be used as a novel biomarker for TB.

## Materials and Methods

### Bacteria, macrophages and infection

Human monocytes were obtained from healthy blood donors (*Etablissement Français du Sang*, EFS, Toulouse, France) with written informed consent (under EFS Contract n°121/PVNT/TOU/IPBS01/2009-0052, which was approved by the French Ministry of Science and Technology, agreement n°AC2009-921, following articles L1243-4 and R1243-61 of the French Public Health Code). Monocytes were prepared following a previously published procedure [15]. For differentiation in macrophages, monocytes were allowed to adhere to the microscope cover glasses (VWR international) in 6-well or 24-well plates (Thermo Scientific), at 1.5x10^6^ cells per well and 3x10^5^ cells per well, respectively, for 2 h at 37°C in warm RPMI 1640 medium (GIBCO). The medium was then supplemented with 10% FCS (PAN-BIOTECH), 1% sodium pyruvate (GIBCO) and 0.1% β-mercaptoethanol (GIBCO), considered from here as ‘complete’ RPMI, and human M-CSF (Miltenyi Biotec) at 20 ng/mL. Cells were allowed to differentiate for 5–7 days. The cell medium was renewed every third or fourth day of culture. Mouse bone marrow macrophages were prepared as described previously [16].

*M*. *tuberculosis* (H37Rv strain) was grown in Middlebrook 7H9 culture medium (Difco) supplemented with 10% albumin-dextrose-catalase (ADC, Difco), 0.05% Tween-80 (Sigma), or on Middlebrook 7H11 agar (Difco) supplemented with 10% oleic acid-ADC (OADC, Difco). GFP-expressing *M*. *tuberculosis* was generated and cultivated as previously described [15]. For *in vitro* infection experiments, bacterial clumps were disaggregated after at least 20 passages through a 25G needle. Macrophages were infected with *M*. *tuberculosis* at a multiplicity of infection of 1 bacterium/10 macrophages in complete RMPI medium for 4 h at 37°C. Cells were then washed in RPMI and further incubated at 37°C for the indicated time periods. For bacterial counting, cells were lysed and cell lystaes were plated onto 7H11 agar medium for CFU scoring.

### Binding and opsonization experiments

All binding experiments were performed in TBS buffer (20 mM Tris, 125 mM NaCl, 2 mM CaCl_2_) containing 0.5 mg/mL BSA (Sigma). Non-specific binding to *M*. *tuberculosis* was prevented by incubating the bacteria for 30 min at room temperature in TBS buffer containing 2 mg/mL BSA. Bacteria were then incubated with native CL-LK (5 μg/mL, prepared as in [17]) at 37°C for 1 h in the presence or absence of 20 mM EDTA (Euromedex) or 50 mg/mL purified mannan. After washing in TBS, bacteria were incubated with a biotinylated monoclonal anti-CL-K1 antibody (HYB14-29 [18]) at 2 μg/ml for 1 h at room temperature. Secondary detection was achieved using APC-coupled Streptavidin (eBioscience) (for 20min at room temperature). Bacteria were then washed and fixed for 2 h at room temperature in PBS containing 4% paraformaldehyde (Polyscience)

### FACS & ELISA analysis

For lipoarabinomannan binding experiments, ManLAM or demannosylated ManLAM (αManLAM, prepared as previously described [19]) were coated in 96-well plates (Nunc Immuno-plates Maxisorp, Sigma) in water:ethanol (1:1, v/v) at 100 ng/well. The plates were dried and incubated with TBS buffer containing 2 mg/ml BSA at room temperature for 2 h (saturation step). The plates were then incubated with different concentration of native CL-LK at room temperature during 2 h, in the presence or absence of EDTA or mannan, as described above. After washing, CL-LK was detected using the biotinylated monoclonal anti-CL-K1 antibody at 0.5 μg/mL (1 h at room temperature) and streptavidin-HRP at 100 ng/mL (30 min at room temperature). After washing, substrate solution (TMB substrate reagent set, BD Biosciences) was added for 30 minutes at room temperature and the reaction was stopped with H_2_SO_4_. Colorimetric analysis was performed using a spectrophotometer at an OD of 450nm-570nm. For opsonization experiments, GFP-expressing *M*. *tuberculosis* H37Rv was incubated with native CL-LK (10 μg/ml) at 37°C for 1 h in presence or absence of 10% AB human serum (Sigma). Human macrophages were then infected at a multiplicity of infection of 5 bacteria/macrophage for 4 h at 37°C under 5% CO_2_ in complete RPMI. Cells were then washed, fixed and analyzed by flow cytometry (LSRII, BD Biosciences).

### Mouse, infection and RT-qPCR

CL-K1^-/-^ mice were generated as described elsewhere (Wakamiya *et al*. Manuscript in preparation). Gene inactivation was verified by PCR after tail DNA extraction using the Fast Tissue-to-PCR kit (Fermentas) following the manufacturer’s instructions. PCR was performed using primers P1 (5’-CTGCTTTCAAGCCATGAATCTCTGTTTGTA-3’), P2 (5’-CAGCAGGGACAGGAAAGCCAGGCTAATCAG-3’) and P3 (5’- CTTGGGTGGAGAGGCTATTCGGCTATGACT-3’) and using the PCR mastermix kit (Fermentas) following the manufacturer’s recommendations. Cycling conditions consisted in 10 min at 95°C, followed by 30 cycles of (0.5 min at 94°C, 1 min at 65°C, 1 min at 72°C), followed by 7 min at 72°C. A typical PCR result is displayed in S1 Fig All mouse experiments were performed in animal facilities that meet all legal requirements in France and by qualified personnel in such a way to minimize discomfort for the animals. All procedures including mouse studies were conducted in strict accordance with French laws and regulations, in compliance with the European community council directive 68/609/EEC guidelines and its implementation in France. All protocols were reviewed and approved by the Regional Ethical Committee (reference MP/ 04/26/07/03). All efforts were made to minimize suffering. The study did not involve humane endpoints. For colony forming unit (CFU) counts of lungs and spleens and RNA collection, mice were euthanized by cervical dislocation. Six-to-twelve-week-old female C57BL/6 mice (CL-K1^-/-^ and WT) were anesthetized with a cocktail of ketamine (60 mg/kg; Merial) and xylasine (10 mg/kg; Bayer) and infected intranasally with 1,000 mycobacteria. At different time points, mice were sacrificed, and lungs and spleen were collected, lysed and bacterial CFUs were scored after plating onto agar 7H11 medium. RNA from lungs was extracted using the RNeasy mini kit (Qiagen). The amount and purity of RNA was measured using a NanoDrop ND-1000 apparatus (Thermo Scientific) by measuring absorbance at 260/280nm. Double-stranded cDNA was reverse-transcribed using the M-MLV Reverse Trancriptase kit (Invitrogen), according to the manufacturer’s protocol. Specific PCR primers (S1 Table) were designed using QuantPrime. Real time quantitative PCR was performed with gene targeted primers using qPCR Mastermix plus SYBR Green (Eurogenetec), according to the manufacturer’s protocol. All real-time qPCR reactions were carried out using a 7500 Real-Time PCR System and data were analyzed using the 7500 Software v2.0.6 (Applied Biosystems). PCR array data were calculated by the comparative cycle threshold method, normalized with hypoxanthine-guanine phosphoribosyltransferase (HPRT) housekeeping gene, and expressed as mean fold change in experimental samples relative to control samples.

### Patients

Human plasmas were obtained from participants with informed consent of the study that was approved by the national ethics committee to the Ministry of Health in Madagascar (Agreement N°033-SANPF/CAB of the 20 February 2004). TB patients over 15 years of age were recruited at the principal TB health centre in Antananarivo, Madagascar. The household contacts of the included TB patients were visited at home by the study physicians and invited to join the study if they were ≥1 year old, living in the same house as the TB patient. The healthy controls were selected among the healthy consulting individuals at the anti-rabies centre at the Institute Pasteur of Madagascar. Subjects were invited by the physicians to give informed consent, interviewed and examined. Only subjects who gave informed consent were included. Participants underwent a PPD skin test (10 units; Tuberculin Purified Protein Derivative, Aventis Pasteur). Induration was recorded after 72 hours. A venous blood sample was drawn into a Vacutainer tube containing heparin for ELISPOT-Interferon-Gamma Release Assay (IGRA). PBMC were separated from heparinized whole blood and plasmas were stored at -80°C for further analysis. PBMC were purified as described elsewhere [20]. ELISPOT-IGRA was performed as previously described [21]. Wells were counted and the mean number of spot-forming cell (SFC) per well for each antigen was calculated. The mean number of SFC of the negative control was subtracted and transformed to the number of SFC per 2 x10^5^ cells. The cut-off point for IGRA-positivity was taken as 1.64 standard deviations of the negative control for the whole cohort. Patients were treated according to the National TB Control Program strategy, *i*.*e*. 8-month therapy including 2 months with isoniazid, rifampicin, ethambutol and pyrazinamide, followed by 6 months with isoniazid and ethambutol.

### CL-LK (CL-K1) quantification

The concentration of CL-LK was quantified by assessment of the concentration of CL-K1 by ELISA, as previously described [18]. This sandwich ELISA based on two different MAbs anti-CL-K1 gives parallel curves between measurements of CL-K1 and CL-LK [14].

### Statistical analysis

Data were analyzed using the Student’s *t* test. Correlations were evaluated using the Pearson’s test. ROC analysis was performed using the Prism software package.

## Results and Discussion

**Fig 1:**
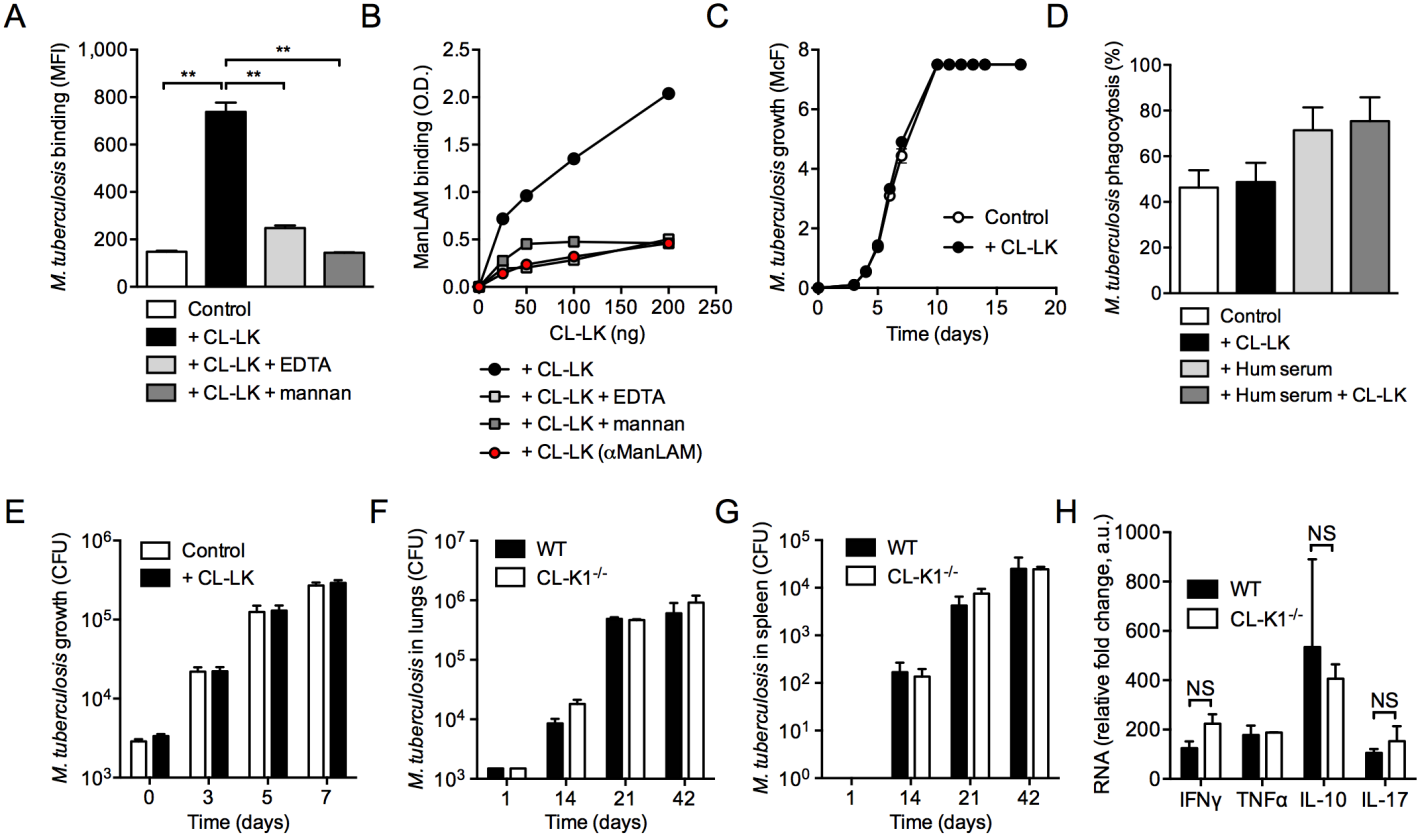
CL-LK recognizes ManLAM in *M*. *tuberculosis*. (**A**) *M*. *tuberculosis* H37Rv was incubated with (+CL-LK) or without (Control) native CL-LK (5 μg/mL) at 37°C for 1 h in the presence or absence of 20 mM EDTA, or 50 mg/mL purified mannan. Bacteria were washed and further incubated with a biotinylated monoclonal anti-CL-LK antibody that was subsequently labeled with APC-coupled streptavidin. Bacteria were analyzed by flow cytometry and the mean fluorescence intensity (MFI) of a triplicate experiment is reported (±s.d.). Data were analyzed using the Student’s *t* test; **, *P*<0.01; ***, *P*<0.001. A representative experiment, out of three independent experiments, is displayed. (**B**) Plastic plates were coated with 100 ng ManLAM or demannosylated ManLAM (αManLAM) per well, and incubated with the indicated concentration of native CL-LK at room temperature for 2 h, in the presence or absence of EDTA or mannan, as in (A). After washing, CL-LK was detected using the biotinylated monoclonal antibody and HRP-coupled streptavidin. Results are obtained by reading OD (450 nm-570 nm) with a spectrophotometer. A representative experiment, out of three independent experiments, is displayed. (**C**) *M*. *tuberculosis* H37Rv was cultured in 7H9 medium enriched with 10% complete human serum, in the presence (+CL-LK) or absence (Control) of 2 μg/mL native CL-LK. Bacterial growth was monitored by turbidity measurement (McFarland units, McF); a representative experiment, out of three independent experiments, is displayed. (**D**) Human monocyte-derived M-CSF-differentiated macrophages were incubated with GFP-expressing *M*. *tuberculosis* H37Rv at a multiplicity of infection of 5 bacteria/cell for 4 h at 37°C under 5% CO_2_ in RPMI containing 10% FCS. Cells were washed and analyzed by flow cytometry. Phagocytosis % represents mean±s.d. of GFP^+^ cells of a triplicate experiment. A representative experiment, out of two independent experiments, is displayed. (**E**) Human macrophages were infected with *M*. *tuberculosis* at a multiplicity of infection of 0.1 bacteria/cell. After 4 h (time-point 0), cells were washed and further incubated in complete medium. At the indicated time-points, cells were lysed in water and bacterial CFUs were scored after plating onto agar 7H11 medium and incubation for three weeks at 37°C. Data show mean±s.d. mycobacterial growth (in CFUs) of three independent experiments. (**F & G**) CL-K1-deficient (CL-K1^-/-^) mice and their wild-type (WT) littermates were infected intranasally with 10^3^ *M*. *tuberculosis* CFUs. At the indicated time-points, lungs (F) and spleen (G) were collected, lysed and bacterial CFUs were scored after plating onto agar. Data show mean±s.d. mycobacterial growth (in CFUs) of one experiment (n = 5) representative of two independent experiments. (**H**) Relative RNA expression for the indicated cytokines were quantified from the lungs of infected mice 21 days after infection, compared to infected WT mice from 14 days. Data show mean±s.d. ΔΔC_T_, compared to HPRT housekeeping gene. The reported experiment is representative of two independent experiments. Data were analyzed using the Student’s *t* test; NS, not significant.

In order to evaluate whether CL-LK binds to *M*. *tuberculosis in vitro*, we conducted a binding experiment where bacteria were incubated with serum-purified human CL-LK for 1 h at 37°C. CL-LK was subsequently detected using secondary fluorescent staining and bacteria were analyzed by flow cytometry. Our gating criteria for flow cytometry analysis are detailed in S2 Fig Our data (Fig 1A & S2 Fig) indicate that CL-LK is able to bind *M*. *tuberculosis* in a dose-dependent manner, and that this binding is Ca^2+^- and mannose-dependent, as EDTA and mannan could inhibit this process. By contrast *M*. *tuberculosis* binding by commercially available recombinant CL-K1 was inefficient (data not shown), most likely because of perturbed oligomerization, compared to the native form. The lipoglycan mannose-capped lipoarabinomannan (ManLAM) is one of the major constituents of the mycobacterial cell envelope, and is recognized by several C-type lectins, such as SP-A, SP-D, MR and DC-SIGN [15,22]. *In vitro* binding experiments indicated that ManLAM is recognized by CL-LK, in a Ca^2+^- and mannose-dependent manner (Fig 1B). Moreover ManLAM recognition by CL-LK relies on mannose caps in ManLAM, as their removal upon treatment with an α-mannosidase resulted in severe reduction in lipoglycan binding by CL-LK (Fig 1B). Interestingly, we found that CL-LK does not bind to the fast growing mycobacterial species *Mycobacterium smegmatis*, in which the LAM molecule is devoid of mannose caps (data not shown).

*M*. *tuberculosis* has been reported to activate the complement cascade through the classical, lectin and alternative pathways, which was shown to promote mycobacterial opsonization with complement components, and to enhance mycobacterial phagocytosis by host macrophages [23,24]. Various collectins are able to inhibit bacterial growth through binding and subsequent triggering of the complement activation cascade, leading to the formation of the membrane attack complex (MAC). Whether this holds true for *M*. *tuberculosis* is still unknown [25]. Nevertheless we decided to assess whether MAC activation and mycobacterial killing could be a functional consequence of CL-LK recognition of *M*. *tuberculosis*. However, the *M*. *tuberculosis* growth *in vitro* was not affected by incubation with CL-LK in the presence of 10% complete complement-containing human serum (Fig 1C). Another function for collectins, such as SP-A and-D, is to modulate the bacterial binding, phagocytosis and trafficking in host macrophages [3]. Yet, the infection of human macrophages by CL-LK-opsonized *M*. *tuberculosis* did not result in altered phagocytosis and intracellular survival of the pathogen (Fig 1D and 1E). In order to evaluate whether CL-LK has an impact on *M*. *tuberculosis* virulence and immune response to infection *in vivo*, CL-K1-deficient mice and their wild-type counterparts were infected intranasally with 10^3^ CFUs *M*. *tuberculosis*, and infection was monitored in the lungs and spleen of the infected animals. Our data indicate that CL-K1 (and thus CL-LK) deficiency has no impact on organ colonization by the pathogen (Fig 1F and 1G), which correlated with similar inflammatory response in the lung, as measured by IFNγ, TNF-α, IL-10 and IL-17 transcripts (Fig 1H). In addition, *in vitro* we found that TNF-α, IL-10 and IL-6 production by *M*. *tuberculosis*-infected wild-type or CL-K1-deficient mouse bone marrow-derived macrophages was similar, and that M. tuberculosis multiplies equally well in wild-type and CL-K1-KO macrophages (data not shown). Altogether, these data indicate that although CL-LK can recognize *M*. *tuberculosis in vitro*, it does not appear to have an *in vitro* functional consequence nor does it play a major *in vivo* role in immune defense against the bacillus, at least in the mouse model.

**Fig 2:**
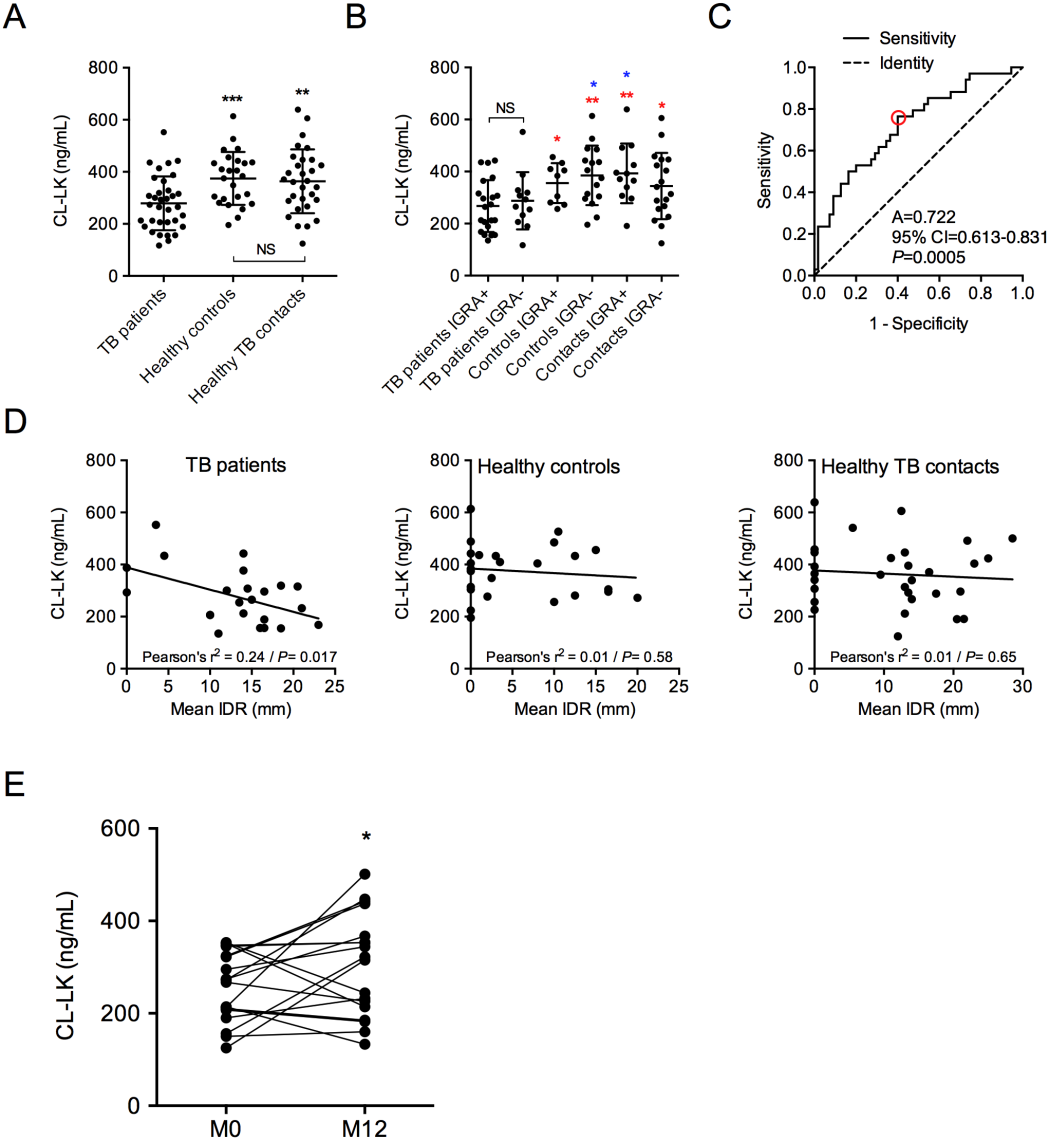
CL-LK is diminished in the serum of patients with TB. CL-LK was quantified by ELISA in the serum of TB patients (n = 34), healthy controls (n = 25) and healthy TB patients’ contacts (n = 30). (**A**) CL-LK concentration according to health status. (**B**) CL-LK concentration according to health status and IGRA status. (**C**) ROC curves of CL-LK concentration in TB patients and healthy individuals. The red circle indicates best detection threshold (334 ng/mL) with a sensitivity of 0.76 and a specificity of 0.6. A, area under the curve; CI, confidence interval. (**D**) Correlation analysis between CL-LK concentration in the serum and mean IDR size (mm) in TB patients (left), healthy controls (center) and healthy TB patients’ contact individuals (right). **(E)** CL-LK concentration in TB patients before (M0) and after (M12) 12 months of treatment. In (A), (B) and (E), data were analyzed using the Student’s *t* test; *, *P*<0.05; **, *P*<0.01; ***, *P*<0.001; NS, not significant. In (B) red stars indicate comparison with IGRA+ TB patients, blue stars indicate comparison with IGRA- TB Patients.

We next decided to quantify CL-LK (CL-K1 subunit) concentration in the serum of HIV-negative patients with TB, as well as in the serum of healthy contacts and control individuals. The whole set of data is available in S2 Table. CL-LK concentration in the serum was correlated neither to gender (356.3±99.09 ng/mL in healthy control males *vs*. 379.7±124.4 ng/mL in healthy control females), nor to age (Pearson r^2^ = 0.03, *P* = 0.19, in healthy controls), in agreement with our previous report [26]. Surprisingly, the amount of CL-LK in the serum of patients with active TB was found significantly reduced (278.8±103.4 ng/mL), compared to that of healthy controls (374.6±101.7 ng/mL, *P* = 0.0008) and healthy TB contacts (363.7±122.7 ng/mL, *P* = 0.0039) (Fig 2A). Interferon-gamma release assay (IGRA) positivity reflects previous exposure and immune response to *M*. *tuberculosis* specifically, as the test quantifies T cell response against the *M*. *tuberculosis*-specific antigen ESAT-6, which is present neither in the BCG vaccine strain nor in environmental mycobacteria. IGRA-positive healthy controls and TB contacts are thus considered subjects with latent TB infection, while IGRA-negative healthy controls and TB contacts are both considered non-infected. When we distinguished individuals according to their IGRA status, only IGRA-positive TB patients were at strong variance with all other healthy individuals regarding CL-LK concentration in the serum (Fig 2B). Receiver operating characteristic (ROC) curve was generated (Fig 2C). The area under the curve was 0.722 (*P*<0.001), with an optimal cut point providing a sensitivity of 76%, a specificity of 60%, a positive predictive value (PPV) of 54%, and a negative predictive value (NPV) of 80% at a threshold of 334 ng/mL (red circle in Fig 2C). In TB patients only, CL-LK concentration correlated inversely to the size of intradermal reaction (IDR), which reflects delayed hypersensitivity to mycobacterial antigens (Fig 2D, left panel); this was not observed in healthy contacts and control individuals (Fig 2D, middle and right panels). Finally, CL-LK concentration was partially restored in TB patients after treatment (256.4±75.4 ng/mL before treatment *vs*. 303.2±110.8 ng/mL after treatment, P<0.05, Fig 2E). From this analysis, we conclude that CL-LK concentration in the serum is diminished in patients with active TB, and inversely correlates to the strength of anti-mycobacterial immune response.

All aspects considered, we believe this study makes two major contributions to the TB field. The first is the identification of a novel soluble immune receptor, CL-LK, for *M*. *tuberculosis*. Native CL-LK purified from human serum recognizes mannose caps in the mycobacterial ManLAM molecule; it might also recognize other mannosylated motifs in the *M*. *tuberculosis* cell envelope, which will need to be explored. Although we could not evidence a role for CL-LK in mycobacterial phagocytosis and intracellular survival of the bacillus in macrophages, and although CL-K1-deficient mice do not display any obvious susceptibility phenotype upon *M*. *tuberculosis* challenge, this does not rule out a role for CL-LK in immunity to *M*. *tuberculosis* in man. These findings are reminiscent of previous results obtained with other pulmonary collectins, namely SP-A and SP-D. While these two collectins were shown to influence, to some extent, mycobacterial interactions with host cells *in vitro*, mice deficient for one or both of these molecules were not more susceptible to *M*. *tuberculosis* infection [8]. This is likely explained by i/ potential functional redundancy between collectins and other C-type lectins, such as the MR and DC-SIGN [15,24,27], in immunity to TB; ii/ in particular, the presence and potential functional redundancy of CL-L1 in CL-K1 deficient mice might account for the lack of manifestation of phenotypic changes after *M*. *tuberculosis* infections; iii/ sequence and structural differences between human and mouse collectins (*e*.*g*. although human and mouse CL-K1 sequences share 90% identity on the primary amino acid sequence level, important amino acid differences exist in their collagen-like and C-type lectin domains); iv/ the profoundly different clinical manifestation of *M*. *tuberculosis* infection in mice and human. One way to evaluate whether CL-LK plays a role in TB in human would be to assess whether genetic mutations and splicing variants of CL-K1 [28] are associated to susceptibility to TB.

The second contribution of our study is the newly identification of a novel biomarker, CL-LK concentration in the serum, for TB disease. Our analysis provides unequivocal evidence that CL-LK amount is reduced in the blood of patients with active TB, as compared to healthy controls, and that this CL-LK concentration is inversely correlated to IDR size, which reflects the strength of anti-mycobacterial immune response. Although the predictive value of CL-LK alone is clearly insufficient, it might be worth evaluating whether CL-LK measurement might be part of a biological signature that is indicative for disease severity and useful to monitor treatment efficacy for TB disease. In addition, whether the CL-LK marker is specific to TB should be assessed using cohorts of patients with other diseases, including pulmonary inflammatory disorders.

## Supporting Information

S1 Fig CL-K1^+/+^ and CL-K1^-/-^ mouse genotyping. The KO allele is visualized after amplification with primers P2 and P3. The WT allele is visualized after amplification with primers P1 and P2. The Het allele (+/-) is visualized after amplification with both primer pairs.
(JPG)

S2 Fig CL-LK binding analysis by flow cytometry. **(A)** Bacteria are first gated based on FSC and SSC. **(B)** Bacteria (gate 1, as shown in (A)) are incubated with mock (none), streptavidin-FITC (control), or CL-LK + biotin-conjugated anti-CL-LK antibody + streptavidin-FITC in the presence or not of EDTA or mannan. **(C)** The percentage of FITC-positive bacteria (gate 2 in (B)) is scored. **(D)** Mean fluorescence intensity (MFI) at different CL-LK concentrations.
(TIFF)

S1 Table Primers used for RT-qPCR. The table indicates the sequences for the different forward and reverse primers used in RT-qPCR experiments (HPRT, IFNγ, IL-10, IL-17a, TNF-α).
(XLS)

S2 Table Patients and controls characteristics. The table indicates health status, age, sex, IGRA status, mean IDR size and CL-LK concentration in all analyzed individuals.
(XLS)
