# Supplementary figures and images for: Collectin CL-LK Is a Novel Soluble Pattern Recognition Receptor for Mycobacterium tuberculosis
Source: PLoS One. 2015 Jul 14;10(7):e0132692. doi: 10.1371/journal.pone.0132692 (PMC4501752; doi:10.1371/journal.pone.0132692)

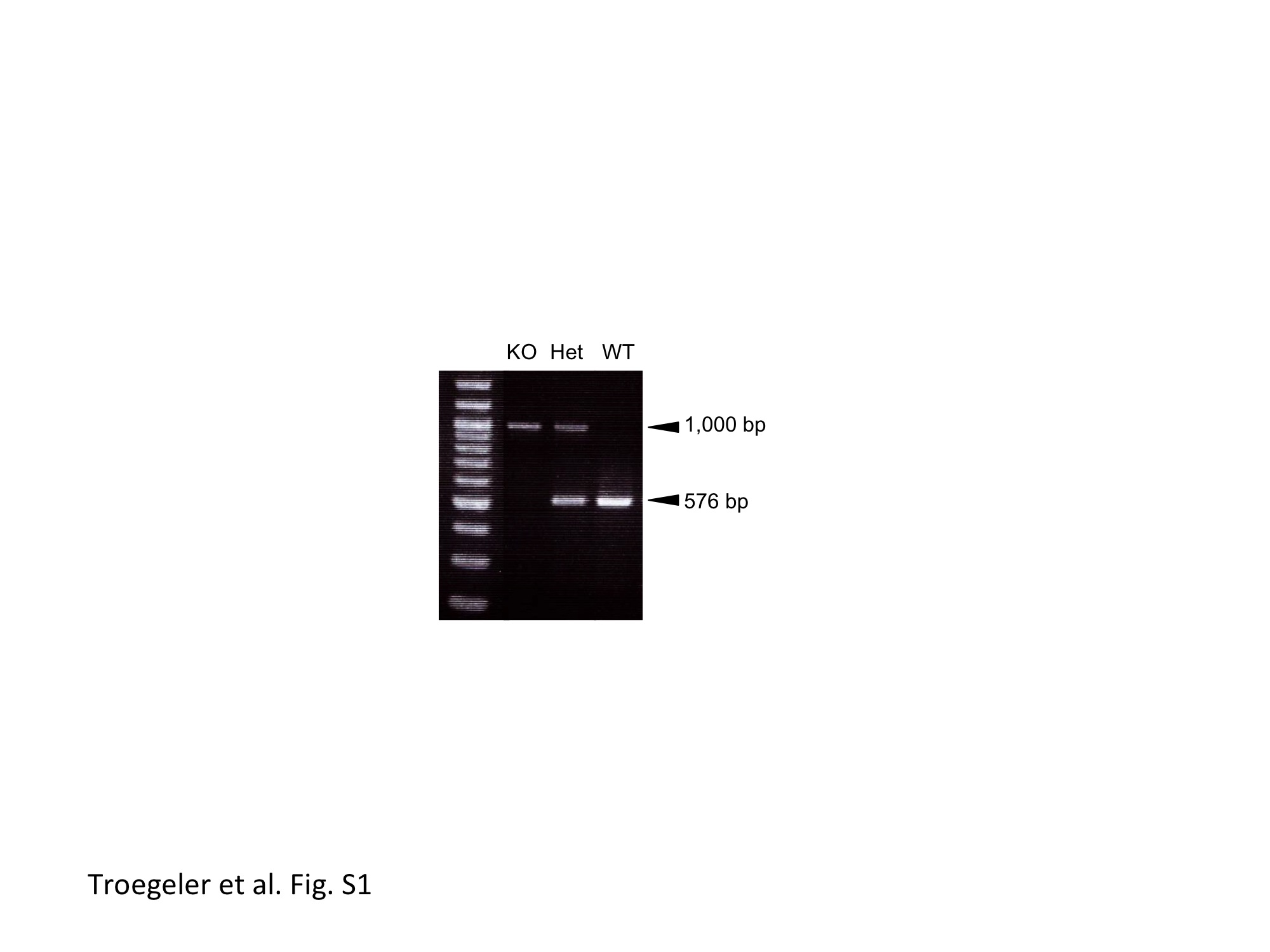

Supplement: S1 Fig — The KO allele is visualized after amplification with primers P2 and P3. The WT allele is visualized after amplification with primers P1 and P2. The Het allele (+/-) is visualized after amplification with both primer pairs. (JPG) [file pone.0132692.s001.jpg]

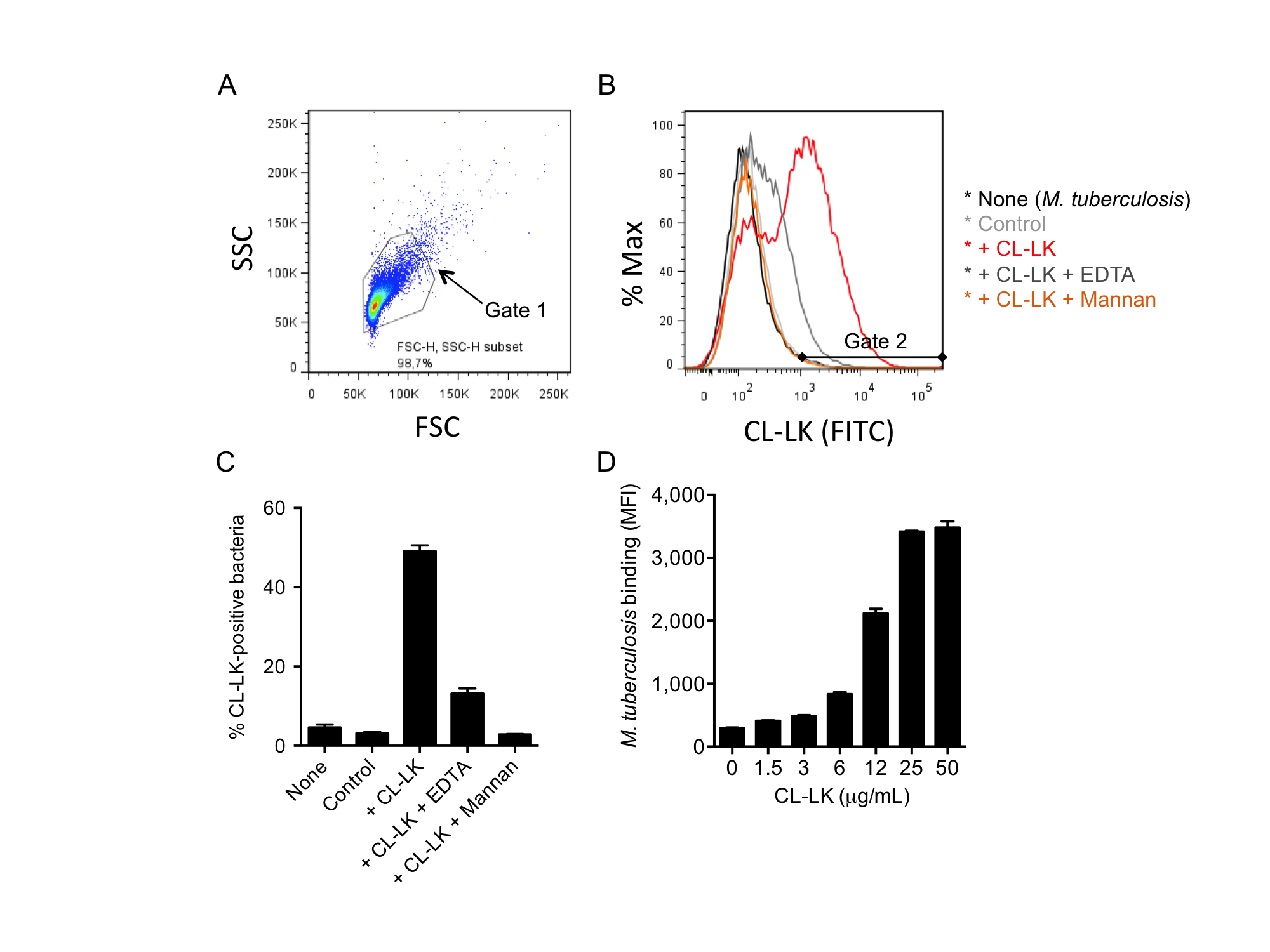

Supplement: S2 Fig — (A) Bacteria are first gated based on FSC and SSC. (B) Bacteria (gate 1, as shown in (A)) are incubated with mock (none), streptavidin-FITC (control), or CL-LK + biotin-conjugated anti-CL-LK antibody + streptavidin-FITC in the presence or not of EDTA or mannan. (C) The percentage of FITC-positive bacteria (gate 2 in (B)) is scored. (D) Mean fluorescence intensity (MFI) at different CL-LK concentrations. (TIFF) [file pone.0132692.s002.tiff]
